# Chitosan Oleate Coated PLGA Nanoparticles as siRNA Drug Delivery System

**DOI:** 10.3390/pharmaceutics13101716

**Published:** 2021-10-17

**Authors:** Dalila Miele, Xin Xia, Laura Catenacci, Milena Sorrenti, Silvia Rossi, Giuseppina Sandri, Franca Ferrari, John J. Rossi, Maria Cristina Bonferoni

**Affiliations:** 1 Department Drug Sciences, University of Pavia, Vle Taramelli 12, 27100 Pavia, Italy; dalila.miele@unipv.it (D.M.); laura.catenacci@unipv.it (L.C.); milena.sorrenti@unipv.it (M.S.); silviastefania.rossi@unipv.it (S.R.); g.sandri@unipv.it (G.S.); franca.ferrari@unipv.it (F.F.); 2 Department of Molecular and Cellular Biology, Beckman Research Institute of City of Hope, 1218 Fifth Avenue, Duarte, CA 91010, USA; xixia@coh.org

**Keywords:** nucleic acid delivery, PLGA nanoparticles, chitosan oleate, siRNA, polyplexes

## Abstract

Oligonucleotide therapeutics such as miRNAs and siRNAs represent a class of molecules developed to modulate gene expression by interfering with ribonucleic acids (RNAs) and protein synthesis. These molecules are characterized by strong instability and easy degradation due to nuclease enzymes. To avoid these drawbacks and ensure efficient delivery to target cells, viral and non-viral vectors are the two main approaches currently employed. Viral vectors are one of the major vehicles in gene therapy; however, the potent immunogenicity and the insertional mutagenesis is a potential issue for the patient. Non-viral vectors, such as polymeric nanocarriers, provide a safer and more efficient delivery of RNA-interfering molecules. The aim of this work is to employ PLGA core nanoparticles shell-coated with chitosan oleate as siRNA carriers. An siRNA targeted on HIV-1, directed against the viral Tat/Rev transcripts was employed as a model. The ionic interaction between the oligonucleotide’s moieties, negatively charged, and the positive surface charges of the chitosan shell was exploited to associate siRNA and nanoparticles. Non-covalent bonds can protect siRNA from nuclease degradation and guarantee a good cell internalization and a fast release of the siRNA into the cytosolic portion, allowing its easy activation.

## 1. Introduction

RNA interference (RNAi) is a natural process occurring in cells to regulate gene expression. Different organisms, from protozoa to mammals, preserved this event during the evolution as a mechanism of defense against viruses [1]. It consists of complex enzymatic machinery able to control post-transcriptional gene expression through the degradation of target messenger RNA (mRNA). RNAi is initiated by double-stranded RNA (dsRNA), which possess a perfectly homologous sequence that can bind the mRNA of the target gene through partial complementarity [2,3,4,5]. In mammalians, the mediators of sequence-specific messenger RNA degradation are 21- and 22-nucleotide small interfering RNAs (siRNAs) generated by the ribonuclease III Dicer and cleavage from longer dsRNAs [6,7]. RNAi can represent a suitable tool for the therapeutic intervention of several human diseases. The key therapeutic advantage of using RNAi lies in its ability to knock down the expression of known sequences specifically and potently of disease-causing genes [8]. In the last years, several research papers and clinical trials highlighted the great potential of RNAi-based therapeutics in cancer treatment, infectious diseases, and other diseases associated with specific gene disorders [4,9,10,11,12,13]. This approach can be useful to treat diseases with “non-druggable” targets for which conventional therapeutics are not successful. Viral infections, ocular diseases, neurodegenerative disorders, and cancer are the most common target, and several preclinical and clinical studies are ongoing [14]. However, gene therapy based on exogenous oligonucleotides, as small interference RNA (siRNA), microRNA, and short hairpin RNA (shRNA), still represent a challenge due to numerous limitations in delivery. As well known, siRNA is rapidly degraded by nucleases, it shows non-specific and poor cellular uptake, and involves low transfection efficiency. To overcome the delivery impediments, many strategies have been proposed. Oligonucleotides can be chemically modified to achieve both better stability and resistance to RNase by attaching different groups at ribose positions such as in 2′-O-methylpurines and 5′-Morfolino modifications [9,15,16]. Alternatively, siRNAs can be encapsulated or conjugated with natural or synthetic delivery vehicles. These include viral or non-viral carriers that can enhance active targeting, involving cell internalization in specific tissues and limiting harmful side effects. The encapsulation can also avoid serum degradation and macrophage phagocytosis [17]. Regardless of the effectiveness of gene transfer, the use of viral vectors presents certain disadvantages. For instance, viruses are immunogenic, and when in the host, may induce an immune response, thus limiting the possibility of repetitive or strengthening dosing and the practical application of gene perturbation in gene therapy [18]. Moreover, the viral delivery itself has been considered nonspecific toward host cell types [19]. Among non-viral vectors, due to their unique properties, such as nanoscale sizes (10–1000 nm), low toxicity, and versatility, nanoparticles have been widely investigated and applied either as drug carriers to treat diseases, and more recently, as a great promising approach to the delivery of novel gene therapeutic agents including siRNA and microRNA. Nanoparticles can help overcoming limits in RNA stability and cellular uptake [20,21,22,23]. Such delivery systems include inorganic nanoparticles (gold or silver nanoparticles) [24,25], lipid-based systems (liposomes, lipoplex and numerous lipid lipid-like materials) [26,27,28], and polymer-based nanostructures [29,30,31]. Nanoparticles based on cationic polymers, for example, can be useful as transfection agents, due to their ability to bind one or more large nucleic acids units, reversibly, into or onto nanoparticles protecting them against bioenvironment degradation. Synthetic cationic polymers include polyethyleneimine and poly-lysine, while natural polymers include chitosan, collagen, and cationic polypeptides [3]. Cationic polymeric nanoparticles based on chitosan can electrostatically interact with negatively charged siRNA upon simple mixing to form stable, positively charged polyplexes [32]. In particular, the amino and hydroxyl groups present in the chitosan chains facilitate the chemical modification enhancing the possibility of a better polymer-nucleic acid interaction [33]. Positive charges of chitosan rely on primary amino groups protonated at pH below 6, which make this polymer useful for applications under slightly acidic conditions, such as tumoral extracellular environments [34]. Several studies investigate the suitability of siRNA-loaded chitosan nanocarriers for different applications [35,36]. A proposed application in the field of gene silencing involves the local delivery of siRNA. For instance, a novel approach is based on using biocompatible implants hybridized with siRNA-loaded chitosan nanocarriers to promote nerve regeneration and allow local delivery of nanotherapeutics [37,38]. The exploitation of electrostatic forces due to chitosan amino groups for siRNA loading has been proposed since the early literature [39]; however, in recent studies, the relevance of ionizable amino groups at the surface of NPs has been highlighted [40]. As well known, the introduction of hydrophobic modification to chitosan offers several advantages, such as easy binding to cells, enhanced nanoparticle stability in serum, better cellular uptake and protection from degradation, and easier nucleic acid dissociation from chitosan inside cells [38]. Based on all these premises, the aim of the present work was to employ a nanoparticle system based on hydrophobically modified chitosan to vehiculate siRNAs. Chitosan-oleate shell-coated PLGA core nanoparticles were previously synthesized [41,42] and characterized as poorly soluble drug delivery systems [43,44]. Their potential advantages, among chitosan-based siRNA carriers, can be found in easy preparation and potential association of drugs loaded in the PLGA core. A preliminary study was carried out to characterize the ionic interaction between negatively charged siRNAs phosphate groups and positively surface charged exposed by chitosan-oleate amino groups present at the surface of the nanoparticles. Quantification of the surface amino groups available for the interaction with siRNA was performed by LC-SPDP (succinimidyl 6-(3(2-pyridyldithio)propionamido)hexanoate) reagent, a method proposed as reference in the literature for the detection of amino groups available on surfaces for further functionalization [45]. An siRNA targeted on HIV-1 and therefore directed against the viral Tat/Rev transcripts was employed as a model [46], and its interaction with the chitosan oleate coated nanoparticles was confirmed through polyacrylamide gel electrophoresis. Studies were performed to verify the interaction of the loaded NPs on immortal and normal cells lines and to assess cytocompatibility.

## 2. Materials and Methods

### 2.1. Materials

Chitosan LMW (CS) (80% Deacetylation Degree, DD), poly-lactic-glycolic acid (PLGA) (Resomer RG 503H), ethyl acetate, and acetic acid were purchased from Sigma-Aldrich (Italy and USA). Oleic acid (OA) was acquired from Fluka (Milan, Italy). All cell culture products were purchased from GIBCO (Gibco BRL/Life Technologies, a division of Invitrogen, Grand Island, NY, USA), Sigma-Aldrich (Milan, Italy and St. Louis, MO, USA), Thermo Fisher Scientific (Carlsbad, CA, USA) and Promocell (Heidelberg, Germany). Silencer siRNA Labeling Kit (Thermo Fisher Scientific). iScript™ cDNA Synthesis Kit and SsoAdvanced Universal SYBR Green Supermix were acquired from Bio-rad (Hercules, CA, USA). Primers and siRNAs were purchased from Integrated DNA Technologies (IDT, Coralville, IA, USA).

### 2.2. Methods

#### 2.2.1. Preparation of Chitosan Oleate PLGA Nanoparticles (CS-NPs)

Chitosan oleate (CS-OA) was obtained in situ by self-assembly [41,42]. Briefly, chitosan was added to 100 mL of bi-distilled water under magnetic stirring (300 rpm) that was slightly acidified (about pH 4.5) with acetic acid to obtain a 1% *w*/*w* polymer concentration. Then, 50% of chitosan binding sites were functionalized with oleic acid, by adding dropwise a stoichiometric amount of fatty acid, solubilized in acetone, to the chitosan solution. CS-OA salt was obtained and, after acetone evaporation, occurred under stirring overnight, CS-OA was freeze-dried for 48 h. The solid residue was employed to obtain NPs by the solvent evaporation method previously described [43,44] and partially modified. Briefly, CS-OA (1.2 mg/mL) was dispersed in 3 mL of distilled water; 0.025 µL amount of glacial acetic acid was added to obtain a better polymer–water dispersion, and 0.25 mL of ethyl acetate solution containing 24 mg/mL of PLGA was poured in all at once during the emulsification step. This step was carried out at 70% amplitude by means of Q700 Ultrasonic processor (QSonica, Newtown, CT, USA) equipped with a replaceable titanium horn (tip diameter = 12.7 mm). After 5 min, 7 mL of distilled water was added, and further emulsification was carried out for 5 min. Then, ethyl acetate was removed under stirring overnight. The day after, NPs were kept under stirring at 40 °C for about 45 min to remove solvent residues and to guarantee the NPs condensation. The weight loss due to evaporation was determined, and the initial volume (10 mL) was reconstituted with distilled water. Finally, NPs were sonicated for 15 min in an ice bath and centrifuged for 10 min at 3000 rpm.

#### 2.2.2. Anti–Tat/Rev siRNAs

CS-NPs were functionalized with a model siRNA targeted to HIV-1 (siRNAs/CS-NPs). These oligonucleotides are directed against the viral Tat/Rev transcripts and are capable to silence the viral expression via post-transcriptional gene silencing (PTGS). Anti-tat/rev Site I 27-mer siRNAs were chosen as a model for our studies and were constituted by the antisense sequence 5′-UGA UGA GCU CUU CGU CGC UGU CUC CGC dTdT-3′ and the sense sequence 5′-GCG GAG ACA GCG ACG AAG AGC UCA UCA-3′ [46]. The efficacy of this 27-mer Dicer-substrate anti-HIV-1 siRNA (dsiRNA) was yet demonstrated in an aptamer–siRNA complex studied in previous works [47,48,49]. The extended antisense strand (MW = 13,616.2 g/mol), end-labeled or not end-labeled with FITC and the complementary extended sense strand (MW = 12,508.4 g/mol) were slowly thawed in ice bath and later annealed in RNase free water and RNA buffer (Tris-EDTA, buffer solution, Sigma-Aldrich), at 65 °C for 5 min, using the same molar ratio as the corresponding partner strand to form the dsRNAs. Finally, annealed siRNAs were gradually cooled from 37 °C to room temperature and kept in an ice bath until their use. The fluorescent dye-labeled siRNAs (FITC-siRNAs) were generated using the Silencer siRNA Labeling Kit (Thermo Fisher) in accordance with the manufacturer’s instructions.

#### 2.2.3. siRNAs/CS-NPs Functionalization

CS-NPs are generally formulated in acidic conditions (pH = 4.5 to ensure both good colloidal stability and fully positive surface charges). However, because siRNAs are unstable at pH below 5 due to the titration of the nucleic bases polar groups mainly involved in hydrogen bonding, the colloidal nano-system was subjected to a buffer exchange in phosphate buffer (DPBS 1X, pH = 7.4) by the aid of a dialysis bag method. Briefly, 5 mL of colloidal NPs was dialyzed in a tube (cut-off 12–14 kDa dialysis tubes, Ø = 36/32–28, 6 mm, Sigma Aldrich) at room temperature, under magnetic stirring (300 rpm) for 1 h, and then the pH (pH ≈ 6) was checked by pH-meter (Mettler Toledo, La Verne, CA, USA). Afterward, 2 µL of siRNAs end-labeled or not end-labeled with fluorescein isothiocyanate (FITC) at different concentrations was directly poured in 100 µL of NPs and incubated at room temperature for 5 min before further analyses.

#### 2.2.4. Characterization of CS-NPs and SiRNAs/CS-NPs

Different molar concentrations of siRNAs (from 200 up to 1000 nM) were employed to ionically functionalize 100 µL of NPs at a fixed chitosan concentration of 60 µg/mL. Both naked CS-NPs and siRNA/ CS-NPs were characterized by measuring the particle size and the zeta potential at room temperature with a NanoBrook, ZetaPALS (Brookhaven Instruments, Holtsville, NY, USA). Reported values are the average of five runs for nanoparticle size and zeta potential. The size measurements were performed by diluting 100 μL of the sample in 3 mL of distilled water, while the zeta potential measurements were performed by diluting 50 μL of the sample in 1.5 mL of distilled water and setting a count rate per second (Kcps) in the range of 2500–3500.

#### 2.2.5. Qualitative Determination of siRNA-NPs Interaction

##### Gel Electrophoresis

The effective binding between siRNAs phosphate groups and chitosan amino groups was qualitatively determined by running a polyacrylamide gel electrophoresis. A discontinuous gel formed from two polyacrylamide solutions, a small, low-percentage stacking gel (4% *v*/*v*), and a larger portion of gel that normally separates proteins (16% *v*/*v*) was prepared. A master mix made on 50% *v*/*v* glycerol solubilized in DPBS 1X and FTIC labeled siRNA/CS-NPs containing 100 nM of FITC-labeled siRNA was prepared. To evaluate a possible dose-dependent interaction between siRNA and CS–NPs, different concentrations of unlabeled siRNA (from 200 up to 1000 nM) diluted in DPBS 1X were added to a fixed volume of master mix for a total of 30 μL/sample. A 1:6 dilution of loading dye (Bromophenol Blue, Thermo Fisher Scientific) was added to master mix and used as control. All samples were loaded, and electrophoresis was run at a constant voltage of 55 V for 2 h in TBE buffer (ultrapure buffer concentrate 0.89 M Tris Borate + 20 mM sodium EDTA, pH 8.3). The siRNA bands were then visualized under a fluorescent imaging system (Gel imaging workstation, Azure Biosystems–c200, USA) fixing λ_ex_ = 490 nm and λ_em_ = 525 nm. The assay was performed in duplicate.

##### Fluorescence Titration Assay

A titration of FTIC-siRNA binding to CS-NPs was performed by the mean of a spectrofluorometer (Plate reader–Spectra Max ID3, molecular Devices, San Jose, CA, USA) reading the fluorescence intensity of FTIC labeled siRNA at λ_ex_ = 490 nm and λ_em_ = 525 nm. The titration was performed by maintaining FTIC-siRNAs concentration constant at 50, 100 or 400 nM and modulating CS-NPs amounts (from 0 to 1200 µg/mL), calculated on the basis of CS-OA concentrations opportunely diluted in water. Eight replicates for each sample were performed.

##### Quantification of Primary Amine Groups

Quantification of the amino group density available on CS-NPs surface was determined by conjugating naked NPs with LC-SPDP (SPDP Crosslinkers, Thermo Fisher Scientific) [50]. The assay was performed both in accordance with the manufacturer’s instructions and by re-adapting the Noels et al. procedure [45] as follows. Briefly, 1 mL CS-NPs opportunely diluted in DPBS 1X was placed in test tubes containing 1.0 mL of 1 mM LC-SPDP prepared in DPBS 1 X + 10% *v*/*v* DMSO. Three different LC-SPDP:CS ratios were tested (1:1,1:2, 1:3 and 1:4). Samples were kept for 150 min at 40 °C in a shaking bath at 100 rpm. The excess of non-reacted species, including oleic acid and PLGA, was removed by three times solvent extraction mediated in dichloromethane (DCM). The LC-SPDP produces bonds containing disulfide that can be reduced after the addition of reducing agents such as DTT. The reaction causes the release of a pyridin-2-thione group, the concentration of which can be determined by measuring absorbance at 343 nm. The aqueous phase recovered was incubated with 1 mL of 25 mM dithiothreitol (DTT) solution for 1 h at room temperature and avoiding the light. The absorbance of the released 2-pyridinethiol groups was measured and recorded spectrophotometrically (UV–Vis Lamba 25, Perkin Elmer, Milan, Italy) at 343 nm. The molar ratio of LC-SPDP bounded to CS-NPs was calculated by applying the formula reported in Equation (1): three replicates were performed, and the exact molar ratio of reacted LC-SPDP was calculate following the Lambert-Beer law.

(1) $$\frac{\Delta A}{8080}\times \frac{Mw\;CS}{\frac{mg}{ml}CS}=moles\;of\;SPDP\;per\;moles\;of\;chitosan.$$

where MwCS is the molecular weight of the monomeric unit of chitosan, ∆A had an absorbance difference at 343 nm after and before DTT reaction, and 8080 value represented the extinction molar coefficient of piridin-2-tione at 343 nm.

#### 2.2.6. In Vitro Evaluation

- HEPG2 cell line

HepG2 cells, derived from a liver hepatocellular carcinoma of a 15-year-old Caucasian male, were purchased from ATCC. HepG2 cells were grown in a polystyrene flask in complete culture medium consisting of EMEM (Eagle’s minimum essential medium, 1.2 g/L sodium bicarbonate, non-essential amino acid, L-glutamine and sodium pyruvate) supplemented with 10% FBS and PenStrep (100 U/mL penicillin, and 100 µg/mL streptomycin). Cells were cultured in a humidified 5% CO_2_ incubator at 37 °C.

- PBMCs cell line

Peripheral blood mononuclear samples were obtained from healthy donors from the City of Hope National Medical Center. PBMCs were isolated from whole blood by centrifugation in SepMate™ tubes through a Ficoll-Hypaque solution (Histopaque-1077, Sigma-Aldrich). following manufactured protocol. Suspension cells were cultured in RPMI 1610 with 10% *v*/*v* FBS, PenStrep and 100 U/mL interleukin 2. Cells were cultured in a humidified 5% CO_2_ incubator at 37 °C.

##### Internalization and Flow Cytometric Analyses on HepG2 and PBMCs

An amount of 1 × 10^6^ cell/well HepG2 and PBMCs was seeded in 12-well plates (CytoOne^®^, Thermo Fisher Scientific) and grown for 24 h in complete medium (EMEM and RPMI 1640 respectively) at 37 °C in a humidified 5% CO_2_ incubator. Afterward, cells were washed twice with 0.5 mL/well of prewarmed DPBS 1X. For PBMCs, every wash was interspersed with centrifugation steps at 300× *g* for 5 min and supernatant withdrawal. Then, cells were treated with 50 µL of FITC-siRNA/CS-NPs diluted in 950 µL of medium without serum and antibiotics to reach siRNA final concentration from 200 nM up to 1000 nM and CS concentration about 60 µg/mL. Furthermore, PBMCs were treated with the same NPs volume at different CS-OA concentrations (from 12.5 up to 100 µg/mL) and functionalized with a fixed amount, 400 nM of FITC-siRNA. After 24 h of transfection, depending on cell line treated, two procedures were performed:

- HepG2 cells were washed twice with DPBS 1X, then trypsinized with 0.5 mL/well of Trypsin EDTA 1 × solution in HBSS (Irvine Scientific, Santa Ana, CA, USA), collected in tubes and centrifuged at 160× *g* for 5 min. Pellets were washed in DPBS 1 X for two times. Finally, cells were fixed with 0.4 mL/tube with IC fixation buffer (Invitrogen, Carlsbad CA, CA, USA) diluted 1:1 in cold DPBS 1× (4 °C). Samples were stored in the fridge, protected from light until FACS (fluorescence activated cell sorting) analysis.
- PBMCs were mechanically detached from the bottom of the well, collected in tubes, centrifuged at 300× *g* for 5 min, the supernatant discarded, and cells were washed once in cold DPBS 1× (4 °C), followed by another centrifuge step. Then, PBMCs were fixed using the same procedure described above.

Internalization was assessed on cells by flow cytometric analysis (FACS) using FACS BD Fortessa (BD Biosciences, San Jose, CA, USA) and FlowJo software version 8.8.6. For each sample, 10,000 gated events were counted and a dot plot of forward scatter versus side scatter established a collection gate (FSC/SSC) for cells to exclude cellular debris, dead and aggregated cells. Gating excluded events with low FSC and high SSC. Fluorescein isothiocyanate (FITC) gate was set using fluorescence minus one control, where cells were not stained, and the FTIC signal intensity was recorded.

##### Cytotoxicity Test on Human CD14+ Monocytes from Peripheral Blood (hMoCD14+-PB) and Morphological Evaluation

Human CD14+ monocytes from peripheral blood (hMoCD14+-PB), single donor cells were seeded in a 96-well plate with a density of 2 × 10^5^ cells/well and grown for 24 h in complete medium (Mononuclear Cell Medium, Promocell, GmbH, D) at 37 °C in a humidified 5% CO_2_ incubator. Afterward, the medium was carefully replaced, and cells treated for another 24 h with 100 µL of CS-NPs diluted in DPBS 1X to reach different CS-OA concentrations (12.5, 25, 50, 75, 100 µg/mL). Then, an MTT test was performed to evaluate the NPs cytotoxicity. Cells were washed once with DPBS 1X; 100 µL of fresh medium and 50 µL of 2.5 mg/mL of MTT (1-(4,5-Dimethylthiazol-2-yl)-3,5-diphenylformazan, Thiazolyl blue formazan, Sigma-Aldrich, Milan, Italy) were added to each well. After three hours, the reagent was withdrawn and 100 µL of DMSO was added to gain the complete solubilization of the purple formazan crystals. Cells’ viability was evaluated by reading the absorbance spectrophotometrically at 570 nm using a FLUOstar^®^ Omega microplate reader (BMG Labtech, Ortenberg, Germany). The morphological evaluation was performed by means of an optical inverted microscope in transmitted light (DMi8S, Leica Microsystem, Milan, Italy). Briefly, 5 × 10^5^ monocytes were seeded in a 24-well plate and treated for 24 h with CS-NPs with a chitosan concentration of 50 and 100 μg/mL. Then, cells were fixed in glutaraldehyde 3% *v*/*v*, and after two hours, were washed twice. The morphology of cells treated with CS-NPs was compared to the cells cultured in growth medium (control).

##### Characterization of Immune Responses of Human PBMCs

To evaluate any immune response from naked CS-NPs on PBMCs, a qRT-PCR was performed. Four pro- and anti-inflammatory cytokines were assessed: IL-6, IL-12, TNFα and INFγ, and 1 × 10^6^ cells, which were cultured for 24 h in a 12-well plate at 37 °C in a humidified 5% CO_2_ incubator. The day after, medium was replaced and cells were treated for another 24 h with 100 µL of NPs diluted in DPBS 1X to reach different CS-OA concentrations (12.5, 25, 50, 100 µg/mL). Finally, PBMCs were washed in DBPS 1X, collected and total RNAs were isolated with TriZol agent (Thermo Fisher Scientific) according to the manufacturer’s instructions. Total RNAs were quantified by using NanoDrop™ (ND-1000, Thermo Fisher Scientific) at 230 nm. cDNA was produced using 1 µg of total RNA. Reverse transcription was carried out using iScript™ cDNA Synthesis Kit (Bio-Rad) according to the manufacturer’s instructions. Expression of the IL-6, IL-12, TNF-α and INF-γ coding RNAs were analyzed by quantitative RT-PCR using SsoAdvanced Universal SYBR Green Supermix (Bio-Rad) and specific primer sets at a final concentration of 400 nM for 50 ng of cDNA. GADPH expression was used for normalization of the qPCR data. Primers were as follows: IL-6 forward primer 5′-CCAGCTATGAACTCCTTCTC-3′; IL-6 reverse primer 5′-GCTTGTTCCTCACATCTCTC-3′.IL-12 forward primer 5’-TGTAAAACGACGGCCAGT-3; IL-12 reverse primer 5’ CAGGAAACAGCTATGACC-3′; TNF-α forward primer 5′-CCG AGG CAG TCA GAT CAT CTT-3′; TNF-α reverse primer 5′AGC TGC CCC TCA GCT TGA-3′; forward primer IFN-γ TGT AGC GGA TAA TGG AAC TCT TTT; reverse primer IFN-γ AAT TTG GCT CTG CAT TAT T. GADPH forward primer 5′-CAT TGA CCT CAA CTA CAT G-3′; GAPDH reverse primer: 5′-TCT CCA TGG TGG TGA AGA C-3′. As indicated by the kit, the thermal cycling program was set as follows: polymerase activation was achieved in 30 s at 95 °C; DNA denaturation at 95 °C for 15 s and annealing at 60 °C for 30 s repeating cycles 39 times. Finally, melt curves were recorded.

## 3. Results and Discussion

### 3.1. Characterization of CS-NPs and siRNA/CS-NPs

CS-OA represents an amphiphilic biopolymer derivative obtained by electrostatic interactions of chitosan with oleic acid. In recent years, this hydrophobically modified chitosan was employed for the synthesis of nanostructures, for its ability to self-assembling in micelle structures, and as a stabilizer of nano-emulsions due to its amphiphilic properties [43,44]. In this work, nanoparticles with a PLGA hydrophobic nucleus and shell-coated with chitosan oleate (CS-NPs) were obtained by the solvent–evaporation method, as previously described [43]. As shown in Table 1, nanoparticles were characterized by particle size in the nanometric range (≈240 nm), and a good polydispersity index PI (≈0.2), indicating nanostructure with optimal physical stability. The strongly positive zeta potential values confirmed the effective presence of protonated chitosan amino groups on nanoparticles surfaces. These results are consistent with the ones reported in previous works [41,42]. The effect of pH on the zeta potential is consistent with that previously observed for chitosan-TPP-siRNA nanoparticles [39].

**Figure 1:**
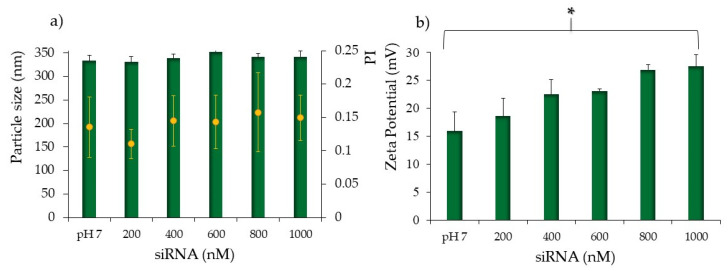
Average particle size (nm, blue bars), and Polidispersion Index (PI, orange dots). (**a**) Zeta potential values (**b**) for the CS-OA NPs in pH7 buffer (control, without siRNA loading) and after the addition of siRNA from 200 to 1000 nM. Analysis of variance (one-way ANOVA) and multiple range test (MRT) (*p* < 0.05); mean values ± s.d., *n* = 5. * indicates statistical significance.

As well known, the high degree of protonation of chitosan amino groups (NH_2_) occurs at a pH below chitosan pKa (∼6.5–6.9) and favors the spontaneous formation of polyelectrolyte complexes through electrostatic interactions with polyanionic molecules such as oligonucleotides [43]. On the basis of this literature evidence, it is conceivable to obtain a spontaneous ionic interaction between siRNA phosphate, negatively charged and CS amino protonated groups at the surface of the CS-NPs, similar to chitosan-based nanoparticles [39,51]. Colloidal NPs are synthesized in an acidic environment (pH 4.5); however, siRNAs are unstable at pH below 5, and to avoid any possible oligonucleotide instability during their complexation with CS-NPs, the colloidal system was subjected to a buffer exchange with the aid of a dialysis bag method. After dialysis, the colloidal stability was checked by particle size, polydispersity index, and surface charges (Table 1). Results showed a statistically significant increase in particle dimensions, while the polydispersity index was unaffected, indicating the maintenance of the good stability of the colloidal system at pH up to 7.4. The positive surface charge of the nanoparticles was preserved, although a drop in the zeta potential occurred. Anionic DPBS phosphate groups, conceivably arranged around the protonated amino groups, neutralizing the positive charge and partially reducing the repulsion forces involved at the shear plane. This led to a minimization of the interparticle distances, causing flocculation phenomena that justify the size increase. Nevertheless, a positive potential still characterizes the nanoparticles, allowing a good binding capacity of chitosan protonated amines to form small and stable siRNAs-polyplexes. Moreover, based on the literature data, a pH slightly lower than 7 is optimal to achieve a good balance between oligonucleotide association and dissociation [52]. As shown in Figure 1a, the addition of siRNAs did not significantly alter the size and colloidal stability, thus this parameter was independent of the entity of the amino/phosphate ratios N/P. On the other side, a slight although statistically significant increase in zeta potential values was evident, showing a shift from ≈15 mV of naked CS-NPs to potential values of ≈28 mV with the highest concentration of siRNAs (1000 nM) (Figure 1b). This can be explained with a slight shift of pH after the addition of more siRNA [39]. The positive values of zeta potential seem consistent with what was previously observed for nucleic acids and chitosan NPs, where negative zeta potential values were observed for nucleic acid excess only, when N/P becomes lower than 1 [53]. In the present case, for all the samples, the N/P ratio is higher than 1, ranging from about 18 to about 4 for the samples loaded with 200 and 1000 nM, respectively. Several factors affect the formation, the structure, and the stability of polyplexes, including: the degree of ionization of polyelectrolytes, their charge density and the polymer chains distribution, the flexibility of the polymer chains, the nature of the ionic groups on the polymer chains, the polyelectrolyte concentration, the mixing ratio, the molecular weights of the polyelectrolytes, the interaction time and temperature and ionic strength, as well as the pH of the medium [54]. Supposedly, siRNAs interposed their structures among the DPBS phosphate groups and the chitosan amino groups, causing a masking effect of phosphate groups of siRNAs that react with chitosan amino groups, while base moieties are exposed with possible variation in charge distribution. This seems consistent with the fact that charge rearrangement looked proportional to the number of reacting siRNAs. However, it is insignificant that it does not affect the inter-particle distances; hence, no particle aggregation and increase in dimensions occurred. Nevertheless, the net positive charge of polyplexes is desirable to prevent particle aggregation and to promote electrostatic interaction with the overall negative charge of the cell membrane [55].

### 3.2. Qualitative Determination of siRNA-NPs Interaction

#### 3.2.1. Gel Electrophoresis

**Figure 2:**
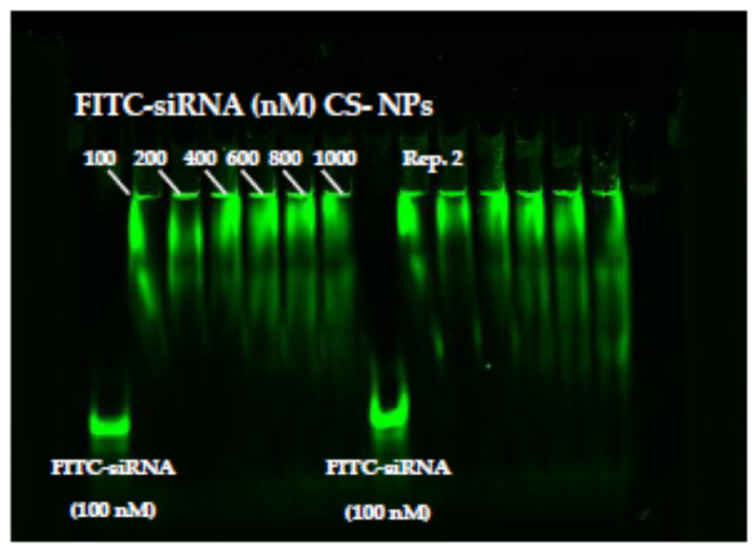
Polyacrylamide gel electrophoresis behavior of FITC siRNA and FITC siRNA loaded on CS-NPs at different concentrations from 100 to 1000 nM. Rep 2 indicates a second replicate of the same experiment.

Figure 2 illustrates the interaction between siRNA’s phosphate group and chitosan’s amino group investigated by gel-electrophoresis analysis. In the Figure, it is possible to see the results of two replicates. FITC-labeled siRNA was employed as a tracking agent, and the binding of siRNA at different concentrations with CS-NPs was observed. The comparison with FITC-siRNA shows a clear holdback in the FITC-siRNA signal that occurred when the oligonucleotides were complexed with NPs. Retention within the well is attributable to positive charge and steric hindrance to diffusion inside the acrylamide gel, which can play a relevant role for NPs that cannot penetrate inside the gel network. Moreover, no apparent differences depending on siRNA concentration employed were evident. The reason for the complexes immobilization during the electrophoretic run was mainly related to the quantity of NP positive surface charge, which in this experiment can be considered constant. The similar behavior observed for all the samples suggests that saturation of the CS-NPs interaction sites was not reached with the highest siRNA concentrations. In no case can a sharp band corresponding to the free FITC siRNA be seen. The siRNA migration patterns also indicate the strength of its interaction with the NPs and the presence of smeared bands suggested that the interaction between chitosan and siRNA obtained by ionic adsorption was weak such that a slow detachment of siRNA under the electrical field occurs. These results were in accordance with previous studies showing that binding was stronger in carriers where siRNAs were at least partially encapsulated in nanoparticles than in carriers where they were on the surface of the cargo, as in the case of simple complexation [54]. The reversibility of the interaction represents an advantage when siRNA must be released by the nanoparticles after cell internalization.

#### 3.2.2. Fluorescence Titration Assay

**Figure 3:**
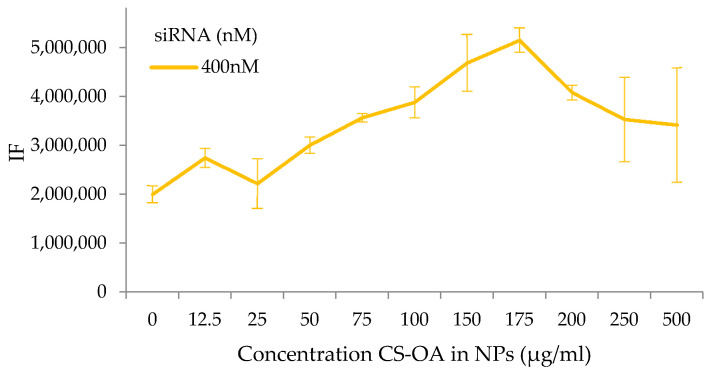
Fluorescence intensity of FITC-siRNA at different concentration of CS-NPs (mean values ± s.d., *n* = 8) for siRNA concentration of 400 nM.

The complex formation was also studied by a binding titration assay. The test was performed by adding a fixed amount of siRNAs (from 50 to 400 nM) to different concentrations of NPs. The different NP concentrations were obtained by progressively diluting the colloidal system in water before the contact with siRNA, and the concentration was calculated considering the quantity of CS-OA used in the preparation. Figure 3 shows in all cases an increase in FTIC-siRNA signal, including with the minimum amount of CS-NPs (CS-OA at 12.5 µg/mL), and a proportional increase in fluorescence with the increase of NPs concentrations was recorded. The increase in fluorescence seems to be related to the growing number of primary amine groups exposed on the surface of the NPs, and therefore arranged in a useful position to interact with siRNAs of about 175 µg/mL of chitosan-oleate. It is possible to argue that this value indicates a quantity ratio between SiRNA and chitosan corresponding to a change in their interaction behavior. At the highest NP concentrations, no further increase of fluorescence occurs, and a plateau or slight decrease can be observed. This can be explained considering that NP surfaces and amino groups available become in excess with respect to siRNA present in the reaction environment. The calculation of N/P ratio for 400 nM siRNA and 175 µg/mL of CS-OA results in a high value of about 27. To better understand this result, a measurement of the ammino groups exposed by the chitosan around the NPs was performed.

#### 3.2.3. Quantification of Primary Amine Groups

**Figure 4:**
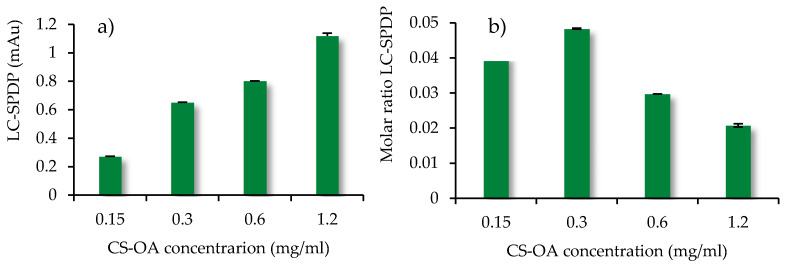
(**a**) pyridine-2-thione absorbance (mAu) at 343 nm released after DTT reaction; (**b**) molar ratio LC-SPDP to chitosan concentration (mean values ± s.d., *n* = 3).

The quantification of the amino group density available on the surface of the CS-NPs was performed by NPs conjugation with LC-SPDP (SPDP Crosslinker, Thermo Fisher scientific). The LC-SPDP reagent is a heterobifunctional crosslinker with two reactive groups, an amino groups and a sulfhydryl group. As indicated in the protocol [35], the reactive portion with amine of the SPDP reagents is the *N*-hydroxysuccinimide ester (NHS), while the sulfhydryl reactive part of the SPDP reagents is the 2-pyridyldithium group, which reacts optimally with sulfhydryl groups between pH 7 and 8.1. The LC-SPDP concentration equal to 1 mM was chosen on the basis of the literature [51] and of preliminary experiments to ensure an excess of the reagent, which covalently binds to all the amino groups present on the surface of the chitosan-coated nanoparticles. Figure 4a shows the absorbance values of the pyridine-2-thione released after dithiothreitol (DTT) reaction, as described in the Methods section. The quantity of pyridine-2-thione released is expected to be proportional to the number of free amino groups exposed on the nano-system surface, and the results obtained were effectively consistent with this hypothesis. The results displayed a progressive increase in absorbance with the increasing of NPs concentration and of chitosan amount at their surface, and therefore of the amino groups available for interaction with the ligand. The molar ratio between LC-SPDP and nanoparticles slightly increases up to chitosan concentration of 0.3 mg/mL, to decrease for further growth of chitosan concentrations. This can be related to the aggregation phenomenon which can occur in the colloidal system, especially at the higher concentrations. This hypothesis is based on previous experience with analogous systems, for which a trend of dimension increase was seen with the increase of concentration [44,56]. The aggregation phenomenon can be responsible for the incomplete exposure of the total free amino groups on the nano-system surface available for the binding with the ligand. At the same time, it must be considered that an aggregative phenomenon can be associated with a change in ionic strength when the system is exposed to the reactive environment. Further studies are needed to better investigate these aspects. However, it can be considered that the quantification of amino groups was adequately estimated on the basis of the highest values obtained at the lowest chitosan concentrations, which indicated a molar ratio between available amino groups and *N*-acetylgalactosamine units of chitosan equal to 0.04–0.05 (Figure 4b). This low ratio can be explained partially with the presence of oleic acid moieties that are used in CS-OA in an amount useful to theoretically interact with 50% of chitosan amino groups, and partially with the mechanism of self-assembly of chitosan chains, that during folding, can cause some amino groups to hinder inside the polymer coils. In light of this result, the high value of N/P ratio calculated by the change of fluorescence in Figure 3 can be interpreted as leading to a ratio closer to 1 if only the surface amino groups are considered. All these findings seem to suggest the occurrence of a cloud of siRNA molecules around the chitosan-coated and positively charged NPs, with a portion of siRNA molecules more directly interacting with the chitosan amino groups exposed at the surface.

### 3.3. Internalization and Flow Cytometric Analyses on HepG2 and PBMCs

**Figure 5:**
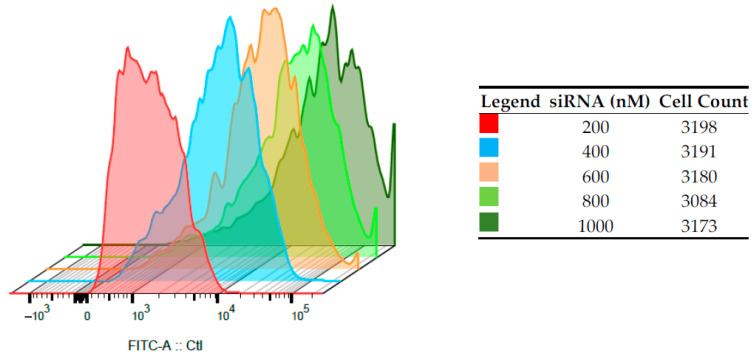
Intracellular fluorescence intensities on HepG2 of FITC-siRNA/CS-NPs complexes prepared at different siRNAs concentrations, determined by flow cytometry (*n* = 3).

**Figure 6:**
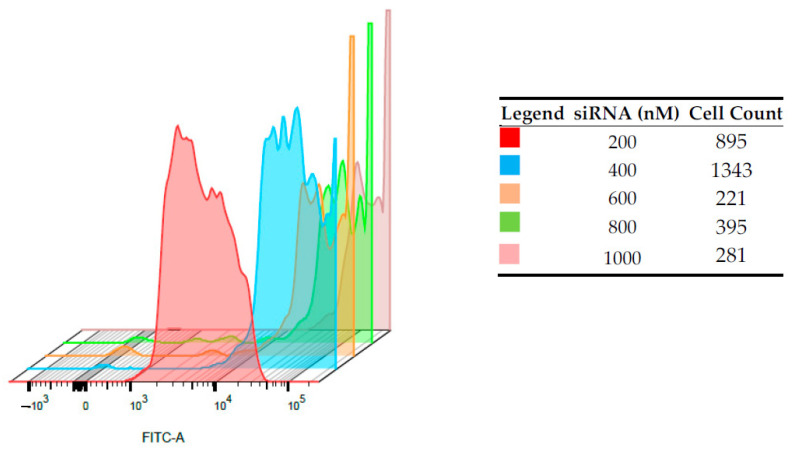
Intracellular fluorescence intensities on PBMCs of FITC-siRNA/CS-NPs complexes prepared at different siRNAs concentrations, determined by flow cytometry.

A fixed amount of CS-NPs (60 µg/mL) was complexed with increasing amounts of FITC-siRNAs, and cell-internalization studies were performed by FACS. The entity of internalization of the complex was determined by detecting the amount of FITC-siRNA taken up by the cells, after 24 h of transfection, in a flow cytometry test. Immortalized HepG2 (Figure 5) and normal human PBMCs (Figure 6) were employed, and in both cases, the increase of the shift and the intensity of fluorescein signal in alive cells was proportional to the amount of siRNA loaded onto the nano-system. For HepG2, a shift indicating higher internalization can be seen with the increase in siRNA concentrations. For example, the FITC intensity values close at about 10^4^ in the case of siRNA 200 nM and at about 10^5^ for siRNA 400 nM. In the case of PBMCs, despite of the relatively low number of cells counted, this shift is more marked, and the more concentrated samples correspond to curves that present intensities higher than 10^5^. This can suggest a positive internalization of the FITC-SiRNA in these cells. the difference between the two cell lines can be observed for the sample loaded with siRNA 200 nm, for which the curve maximum is at intensity slightly lower than 10^3^ for HepG2 and between 10^3^ and 10^4^ for PBMC cells.

### 3.4. Cytotoxicity Test on Human CD14+ Monocytes from Peripheral Blood

**Figure 7:**
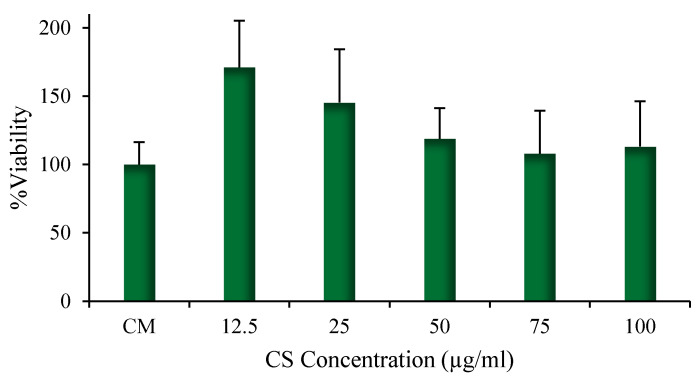
Viability of hMoCD14+-PB cells after 24 h contact with different NP amounts, expressed as CS-OA concentration (mean values ± s.d., *n* = 8).

**Figure 8:**
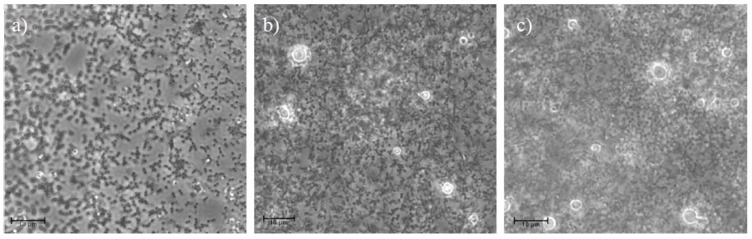
Photomicrographs of the hMoCD14+-PB cells: (**a**) not exposed to CS-NPs (control) and of cells after 24 h of contact with CS-NPs at 50 (**b**) and at 100 µg/mL (**c**) of CS-OA. Scale bar = 10 μm.

The results of the cytotoxicity test performed on human CD14+ monocytes from peripheral blood (hMoCD14+-PB) cells are given in Figure 7. The results demonstrated that the presence of CS-NPs, including at the highest concentration of CS-OA (100 μg/mL), did not affect cell viability. Modification of monocytes toward M1 macrophage phenotype is physiologically induced during infections and results in release of pro-inflammatory factors, while M2 phenotype is characterized by secretion, especially of anti-inflammatory factors. Occurrence of macrophages from monocytes can be appreciated by microscope analysis and was studied in the present case by observing the change in morphology of hMoCD14+-PB cells after exposure to CS-NPs at 50 µg/mL and at 100 µg/mL. The results are illustrated in Figure 8, where the presence of macrophages deriving from hMoCD14+-PB cells for the effect of the sample at both concentrations can be observed.

### 3.5. Characterization of Inflammatory Response of Human PBMCs on CS-NPs

**Figure 9:**
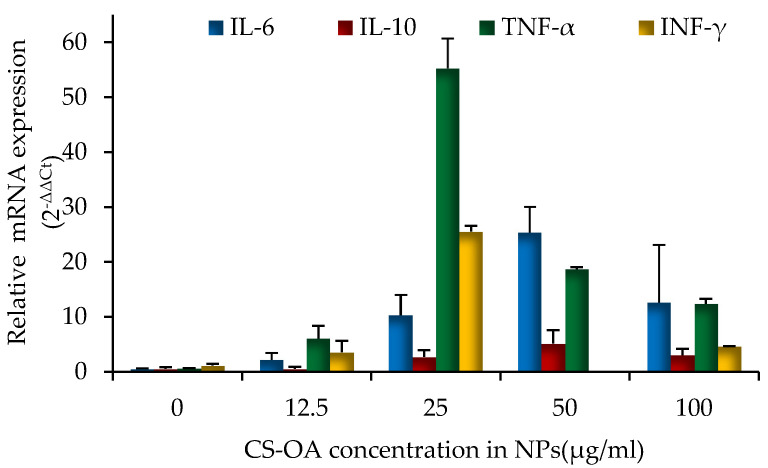
Relative IL-6, IL-10, TNF-α and INF-γ mRNA expression levels (2^−ΔΔCt^). The concentration of CS-NPs is calculated considering the CS-OA content present in the colloidal system (mean values ± s.d., *n* = 8).

The inflammatory response of naked CS-NPs was analyzed on PBMCs with the aid of RT-qPCR technique. The results are illustrated in Figure 9. The concentrations of NPs were calculated considering the CS-OA content present in the colloidal system (from 12.5 µg/mL up to 100 µg/mL) and the cytosolic production of four different cytokines (IL-6, IL-10, TNF-α, and IFN-γ) was evaluated after 24 h of cell exposure to NPs. For both the anti-inflammatory (IL-10) and pro-inflammatory (IL-6, TNFα, INFγ) mediators, a dose-dependent response occurred, reaching the highest level at 25 µg/mL of chitosan concentration in the cell environment. At the highest chitosan concentration, and therefore, nanoparticle amount (50 and 100 µg/mL), cytokines secretion seemed to be lower. As well known in the literature, chitosan has distinct effects on blood coagulation and macrophage activation depending on how chitosan is processed, on molecular weight, deacetylation degree and concentration [57,58]. It has been therefore recognized that chitosan effect can be pro-inflammatory or anti-inflammatory depending on the context [59]. Water-soluble chitosan oligomers and chitosan degradation products, for example, can stimulate both macrophage differentiation and monocyte activation and can involve a dose-dependent secretion of cytokines and mediators of inflammation, including interferon-γ (IFN-γ), tumor necrosis factor-α (TNF-α), interleukin-1 (IL-1), interleukin-2 (IL-2), interleukin-6 (IL-6), nitric oxide (NO) [60]. Given these premises, the pro-inflammatory effect can be related to a nano-system disassembling resulting in the release of chitosan debrides occurring after the NPs endocytosis. A further aspect that should be considered in the present case is the complex composition of the studied NPs, in which chitosan is hydrophobically modified with oleic acid. In addition, for oleic acid such as for other unsaturated fatty acids, both pro-inflammatory and anti-inflammatory activity has been demonstrated [61,62]. Is well known, moreover, that cationic particles induce inflammation to a great extent than anionic and neutral ones [63]. The activation of proinflammatory cytokines IL-6, IL-10, of TNF-α and INF-γ can be bearded by NP surface chemistry and surface charge, which also modulate NP interaction with immune cells [64]. The observed immuno-stimulatory effects suggest avoiding the use of CS-NPs in systemic delivery. Nevertheless, as well known, nanoparticles based on chitosan can be applied in drug delivery systems aimed at oral, local, and topical routes (skin or intact mucosae) [65], possibly exploiting the well-known mucoadhesive properties of the polymer [66]. The association of anti-HIV oligonucleotides to the mucoadhesive and immunostimulatory CS-NPs could be proposed for vaginal delivery to inhibit sexual transmission. It has been demonstrated that siRNAs have great potential in preventing not only HIV but also Herpes and Papillomavirus infections after vaginal delivery [67,68,69]. The use of NP- based non-viral carriers of siRNAs presents several advantages in this case, as an improvement can be found not only in protecting siRNAs from degradation, but also in triggering the interaction with the mucosa and the internalization by the epithelial cells [66]. In this perspective, the immunostimulant activity of the studied CS-NPs can add a beneficial synergic effect against virus infections. This aspect should deserve future investigation. Other applications involving different siRNAs may be aimed for example to local cancer therapy, which is an approach of increasing interest. In this case, the synergic effect of the anti-cancer siRNAs action with the pro-inflammatory activity of CS-NPs may be exploited. To make the effect specific against tumoral cells, NPs can be loaded in biocompatible local drug delivery systems such as implants, 3D scaffolds, or electrospun nanofibers [70], and should be decorated with active targeting ligands [71].

## 4. Conclusions

In previous works, PLGA nanoparticles shell coated with a hydrophobically modified chitosan (CS-NPs) were characterized and studied as drug delivery systems of lipophilic drugs. The present research seemed to confirm the suitability of the same nanoparticles, as non-viral vectors, to be loaded with oligonucleotides, able to regulate the expression of targeted genes and play an active role in fighting specific diseases. In particular, siRNA/CS-NPs polyelectrolyte complexes were obtained. They exploit electrostatic interactions among the positive surface charges of chitosan amino groups and the anionic phosphate groups of siRNAs. The poly-complexes were achieved by simple mixing. The assembly of siRNA onto CS-NPs did not affect the physical stability of the colloidal system: particle size remained constant, and a slight increase in zeta potential values occurred. The complex formation was demonstrated by gel electrophoresis assay. In all the concentration ranges tested, effective although weak interaction was observed among the two components. This appeared independent of the siRNA/CS-NPs ratio. An interaction between NPs and siRNA seemed confirmed by a fluorescence titration test. Furthermore, the cell internalization of siRNA-chitosan NPs complexes was suggested by flow cytometry on two different human cell lines (immortalized, HepG2 and normal, PBMCs). In both cases, the signal of the labeled siRNA present in alive cells was proportional to the siRNA’s concentrations mixed to a fixed amount of CS-NPs. On PBMCs, a dose-dependent pro-inflammatory cytokines production was evident, in particular of TNF-α, and IFN-γ. The exposure of monocytes to CS-NPs induced a macrophage differentiation and activation, possibly due to both chitosan and oleic acid presence in NPs. Even if CS-NPs demonstrate a good versatility as siRNA cargo, the intense immuno-stimulatory effects suggest avoiding their use in systemic delivery. They can however be used for local therapy either for anti-HIV siRNA in vaginal applications or for anti-cancer siRNA in local cancer therapy. Specific studies of course will be needed in this case to highlight the behavior of CS-NPs unloaded and loaded with siRNA in more specific cell lines, such as epithelial cells or fibroblasts. The specific contribution of chitosan and oleic acid, of their concentrations, and of exposure conditions to NP pro-inflammatory or anti-inflammatory effect can also be worthy of further investigation.

## Figures and Tables

**Table 1:** CS-NPs mean particle size (PS, nm), polydispersity index (PI) and zeta potential (ζ mV) (mean ± s.d.; *n* = 3) before and after dialysis.

	Before Dialysis (pH 4.5)	After Dialysis (pH 7.4)
PS (nm)	236.7 ± 4.0	333.6 ± 11.5
PI	0.18 ± 0.03	0.14 ± 0.04
Zeta potential, ζ (mV)	36.49 ± 8.55	15.95 ± 3.40

## Data Availability

The data presented in this study are available on request from the corresponding author.
